# Enhancing Cross-Subject Motor Imagery Classification in EEG-Based Brain–Computer Interfaces by Using Multi-Branch CNN

**DOI:** 10.3390/s23187908

**Published:** 2023-09-15

**Authors:** Radia Rayan Chowdhury, Yar Muhammad, Usman Adeel

**Affiliations:** 1 Department of Computing & Games, School of Computing, Engineering & Digital Technologies, Teesside University, Middlesbrough TS1 3BX, UK; 2 Department of Computer Science, School of Physics, Engineering & Computer Science, University of Hertfordshire, Hatfield AL10 9AB, UK

**Keywords:** convolutional neural network (CNN), brain–computer interface (BCI), deep learning, fusion network, motor imagery (MI), electroencephalography (EEG)

## Abstract

A brain–computer interface (BCI) is a computer-based system that allows for communication between the brain and the outer world, enabling users to interact with computers using neural activity. This brain signal is obtained from electroencephalogram (EEG) signals. A significant obstacle to the development of BCIs based on EEG is the classification of subject-independent motor imagery data since EEG data are very individualized. Deep learning techniques such as the convolutional neural network (CNN) have illustrated their influence on feature extraction to increase classification accuracy. In this paper, we present a multi-branch (five branches) 2D convolutional neural network that employs several hyperparameters for every branch. The proposed model achieved promising results for cross-subject classification and outperformed EEGNet, ShallowConvNet, DeepConvNet, MMCNN, and EEGNet_Fusion on three public datasets. Our proposed model, EEGNet Fusion V2, achieves 89.6% and 87.8% accuracy for the actual and imagined motor activity of the *eegmmidb* dataset and scores of 74.3% and 84.1% for the BCI IV-2a and IV-2b datasets, respectively. However, the proposed model has a bit higher computational cost, i.e., it takes around 3.5 times more computational time per sample than EEGNet_Fusion.

## 1. Introduction

The brain–computer interface (BCI), which decodes brain signals, is a mode of communication between the human brain and electronic devices. BCIs may enable people with low motor strength to connect and interact with their environment without taking physical action. Electroencephalography (EEG) is one of the most popular methods of gathering input from the brain by reading brain waves using a device capable of electrophysiological monitoring of the brain’s electrical activity [1]. The brain’s neural activity is usually recorded using a non-invasive scalp EEG [2]. The global 10–20 standard approach for electrode implantation is used to record brain signals by applying electrodes to the scalp. The terms 10 and 20 denote that the specific gaps between adjacent electrodes account for either 10% or 20% of the overall distance from the front to the back or from the right to the left side of the skull [3,4,5] (from Wikipedia, the free encyclopedia. Available online: https://en.wikipedia.org/wiki/10-20_system_(EEG), accessed date: 5 June 2023). EEG generates readings of electrical potential as time signals for each electrode positioned on the participant’s scalp. These data reflect the brain’s activity and provide vital information that can be used in numerous fields, including cognitive science and medical diagnosis [6]. By utilizing EEG data, researchers can analyze brain functions, explore cognitive processes, identify irregularities or disorders, and potentially assist in diagnosing certain conditions.

Multiple studies have illustrated the connections between EEG signals and both actual and imagined movements [7]. Motor imagery (MI) involves a dynamic mental state where an individual mentally simulates a physical action. MI has gained considerable research attention due to its unique ability to generate brain signals without reliance on external triggers [8].

Recent studies have investigated the application of deep learning to EEG signals. Deep learning (DL) is a type of machine learning that has become popular in recent years. DL techniques have a high learning capacity and can retrieve complicated and significant features from different data sorts. DL models achieved impressive results in EEG motor imagery classification [9,10,11].

EEG-based BCI development has seen widespread use in medical applications. BCI in neurorehabilitation offers promising avenues to enhance recovery and regain function for individuals with neurological conditions and injuries. BCI applications support disabled people to communicate and use assistive technologies like wheelchairs [12,13,14,15], robotic arms [16,17], and wearable lower-limb exoskeletons [18]. BCI has been explored for improving cognitive functions such as stroke neurorehabilitation [19], spinal cord injury, and traumatic brain injury [20]. BCI systems have also been used to provide neurofeedback and cognitive training exercises to enhance attention, memory, and executive functions [21]. Moreover, BCI can contribute to the improvement of neuroplasticity [22], which is the brain’s capacity to modify and adapt its structure and function based on experience [23]. Patients with paralysis, amputations, or central nervous system dysfunction are receiving support through BCI-based prosthetics [24]. Additionally, BCI has been used to assist individuals with locked-in syndrome or amyotrophic lateral sclerosis (ALS) [25,26] and Parkinson’s disease [27]. Imagined speech also has potential in the BCI domain, which allows people with physical disabilities to communicate and use devices by imagining specific commands that the device can then recognize and carry out [28]. This innovative approach holds promise for enhancing the quality of life for patients with physical disabilities.

BCI implementations have also expanded into the entertainment industry, enabling the development of smart environments [29], biofeedback-based games [30], and other forms of entertainment that offer engaging and immersive experiences. In the realm of BCI technology, a study highlights four areas where advancements can bring substantial benefits to disabled individuals: communication and control, motor substitution, entertainment, and motor recovery [2].

BCI applications have become popular due to their affordability, user-friendliness, and adequate temporal resolution [31]. However, the highly discrete pattern of brain signals makes building a cross-subject classifier challenging [32]. Convolutional neural networks (CNN), a type of deep network, have been used in modern studies to explore the possibility of automatic feature extraction. Deep and shallow CNN architectures with distinctive design choices were researched in previous work [33]. EEGNet, a neural network architecture designed for cross-paradigm BCI issues, was first presented in [34]. It can correctly identify EEG signals obtained from diverse BCI systems.

Fusion structures have shown promise in addressing the issue of poor cross-subject data generalization in EEG-based categorization. Fusion networks can extract information from many branches with diverse designs or hyperparameters, combine the features in a fusion layer, and improve the classification accuracy of the architecture [35]. This method is based on the premise that the brain signal patterns of the subjects are unique and that the high-performing networks and hyperparameters can change depending on the subject. Overcoming the challenges of cross-subject classification development will enable the widespread implementation of BCI [36].

A 3D multi-branch CNN with three branches and various strides and kernel sizes in the convolutional layers was investigated in the study reported in [37]. After implementing feature fusion, the softmax classification layer was applied to the final connected layer. On the BCI Competition IV 2a dataset, the model secured 75% accuracy for within-subject classification. Another study [38] applied each pooling layer prior to feature extraction to conduct multi-layer feature fusion. In this study, the features gathered from the four branches were combined, and then the softmax classification layer was added. This method’s accuracy rating on the BCI Competition IV 2a dataset is 74.5%. MMCNN, another multi-branch, multi-scale CNN model, utilized parallel processing with five EEG Inception Networks (EINs) to address subject and time variability issues.

A multi-branch 2D convolutional neural network (CNN) named EEGNet Fusion was introduced in the literature [35] with varying hyperparameters, kernels, and filter sizes for three branches. The primary architecture of each branch follows the EEGNet model [34]. On the *eegmmidb* dataset’s executed movement and imagined movement tasks, this network scored 84.1% and 83.8% accuracy, respectively. The EEGNet Fusion network allowed more freedom in hyperparameter selection and reduced the complexity of subject-independent EEG classification. As a result, this model improved cross-subject classification accuracy.

In the literature, it has been observed that multi-branch fusion networks can improve the accuracy of cross-subject classification in EEG-based BCIs. These networks provide a flexible and adaptable structure that can capture diverse patterns and characteristics across different subjects. Hence, our objective was to examine the performance of higher-branch models and propose an optimal multi-branch model for EEGNet architecture [34]. We aimed to evaluate how the CNN network operates with higher branches and diverse parameter configurations.

In this paper, we propose a novel technique for categorizing motor imagery tasks using a multi-branch feature fusion convolutional neural network model. We improved the EEGNet Fusion model and presented EEGNet Fusion V2, a five-branch convolutional neural network model. We employed various hyperparameters to observe their impact on the model’s performance. Additionally, we implemented four-branch, six-branch, and seven-branch EEGNet Fusion models to evaluate the proposed model. Furthermore, we compared the proposed model, EEGNet Fusion V2, with state-of-the-art models, including DeepConvNet, ShallowConvNet, MMCNN, EEGNet, and EEGNet Fusion.

## 2. Materials and Methods

The dataset used for evaluation, pre-processing steps, proposed 5-branch fusion CNN model architecture EEGNet Fusion V2, and training and testing approach are discussed in this section.

### 2.1. Dataset and Preprocessing

#### 2.1.1. Dataset 1: EEG Motor Movement/Imagery Dataset

Experimental tests of the proposed model was performed using the PhysioNet EEG Motor Movement/Imagery dataset (Available online: https://physionet.org/content/eegmmidb/1.0.0/, accessed date: 22 September 2022) which contains about 1500 one- and two-minute EEG recordings from 109 subjects. The BCI2000 [39] system was used to record EEG data while the experiment’s subjects engaged in various motor imagining activities. Each participant completed 14 experimental runs, which included two baseline runs with their eyes opened and closed as well as three two-minute runs with the tasks of executing or imagining the opening and closing of the left or right hand, both fists or both feet. For the validation of the model, two subgroups of the dataset were used. Left-hand or right-hand movement tasks are included in the first subgroup. Imaginary left-hand or right-hand movement tasks are included in the second subgroup. The motor movement or imagery tasks were recorded as EEG signals on 64 channels placed on subject’s scalps. Each channel is annotated with three codes: T0, T1, and T2. T0 refers to the rest period, while T1 refers to the motion of the left hand in some tasks and both fists in others. T2 denotes the movement of the right hand for some tasks and both feet for others. Each experimental run was partitioned based on these annotations. But according to the literature [35,40], the EEG channels of six subjects (subject 38, 88, 89, 82, 100, and 104) were not annotated as specified in the experiment. As a result, partitioning each experimental run based on these annotations carries the potential risk of making incorrect decisions. Due to incorrect annotations, these six subjects were eliminated. Hence, 103 subjects out of 109 were used.

Each trial’s input data were divided into (C, W) dimensions, where C stands for the number of channels and W is the temporal dimension. All trials contained 4 to 4.1 s sustained and continuous movements for executed and imagined tasks. Therefore, to keep the dataset consistent, 4 s of data were clipped on each trial, sampled at 160 Hz, for a total of 640 samples. The sliding window approach was used to divide the 640 samples into eight non-overlapping windows of 80 samples each. The target label of each window was identical to the initial trial. The eight-time windows can provide more discriminatory information on the motor imagery data. After that, the signal processing module in the Gumpy BCI library [41] was applied to process the EEG signals. To eliminate the alternating current (AC) noise at the 60 Hz frequency, a notch filter was applied to the data. Then, Butterworth band-pass filtering was performed on the data in the 2 Hz to 60 Hz range with an order value of 5.

#### 2.1.2. Dataset 2: BCI Competition IV 2a

BCI Competition IV-2a dataset (BCI Competition IV dataset. Available online: https://www.bbci.de/competition/iv/, accessed date: 15 October 2022) was used to evaluate the proposed model [42]. The Graz University of Technology generated famous public MI-EEG dataset in 2008 known as BCI-2a. The dataset’s small number of samples taken in uncontrolled conditions with many artifacts makes decoding MI tasks difficult. The dataset contains 5184 trials (samples) of MI-EEG data collected from 9 participants applying 22 EEG electrodes (576 trials per participant). Moreover, 3 extra electrooculography (EOG) channels give information about eye movements. MI trials are 4 s long, captured at 250 Hz, and filtered between 0.5 and 100 Hz. Two sessions were captured for each subject on different days. There were 288 trials per subject. Four MI tasks corresponds to each trial: imagined movement of the left hand, right hand, both feet, and tongue.

The time frame length selected for this dataset is 4.5 s (from 1.5 s to 6 s), producing 1125 samples [33]. Standardization was applied in the pre-processing step [43].

#### 2.1.3. Dataset 3: BCI Competition IV 2b

This publicly available dataset [44] was collected from 9 subjects using 3 bipolar electrodes at a sampling rate of 250 Hz. A bandpass filter between 0.5 and 100 Hz was then used for filtering. Moreover, 3 extra EOG channels were employed to collect data on eye movement. The dataset consisted of two classes, called the motor imagery of left hand and right hand. Each subject participated in 2 screening sessions without feedback and 3 screening sessions with feedback. Each session consisted of six runs with ten trials each and two classes of imagery. This resulted in 20 trials per run and 120 trials per session. The pre-processing step was similar to the BCI IV-2a dataset.

### 2.2. Model Architecture

**Figure 1:**
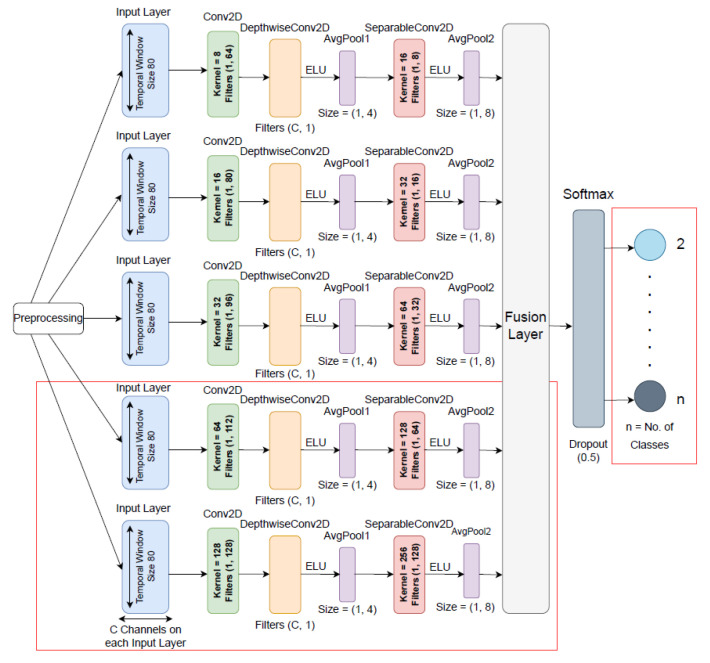
The proposed five-branch fusion convolutional neural network (CNN) architecture, termed EEGNet Fusion V2. This network consists of five branches, and each branch has input layer, a convolutional filter, a depthwise convolutional filter, and a separable convolutional filter. Also, average pooling layers follow the depthwise and separable convolutional filters. Then, the features from five branches are combined using a fusion layer, and the final classification is performed using a softmax activation function in the output layer. Different kernel and filter sizes are used in each branch. This architecture is designed to achieve accurate classification of MI tasks by processing EEG signals. The red box indicates the improvements of the EEGNet Fusion model.

The foundations for the proposed model architecture are the EEG-based CNN model, EEGNet [34], and EEGNet Fusion [35]. The authors in EEGNet [34] demonstrated that any EEG-based classification task may be successfully solved using EEGNet’s original design. The proposed EEGNet Fusion V2 model included five distinct branches, each of the branches received identical input. The architecture of each branch matched with the EEGNet architecture. EEGNet Fusion V2 used different kernel sizes and convolutional filters in the depth-wise and separable layers for all five branches. The fusion method helps to reduce variance and enhance accuracy by aggregating the diverse predictions from different branches. Figure 1 illustrates the proposed EEGNet Fusion V2 architecture.

First, 2-D input with temporal (datapoints per sample) and spatial (number of channels) dimensions are transmitted to the input layer for each sample. The model has five branches, and each branch has a 2-D convolutional layers with kernel sizes 8, 16, 32, 64, and 128, and filters with sizes (1, 64), (1, 80), (1, 96), (1, 112), and (1, 128), respectively. By using the convolutional layers in each branch, the model initially learns frequency filters with temporal convolutions.

After the initial temporal convolutional layer, a depth-wise convolutional layer with (C, 1) filters for all branches was added, where C refers to the number of channels. The depth-wise convolutions help to decrease the number of trainable parameters and extract frequency specific spatial filters for each temporal filter [35].

Batch normalization, a useful technique for minimizing overfitting and enhancing the network’s training pace, was used after the convolutional layer.

After that, a separable convolutional layer was added to each branch with kernel size 16, 32, 64, 128, and 256 and filter size (1, 8), (1, 16), (1, 32), (1, 64), and (1, 128), respectively. To acquire temporal summaries of each feature map independently and to integrate the feature maps, the separable convolution merges pointwise and depth-wise convolutions [34]. Batch normalization was also used after the separable convolutional layer.

An exponential linear unit (ELU) activation function followed each convolution layer. In CNN-based classification, this activation function is more computationally effective [45].

Furthermore, an input representation’s dimensions were down-sampled by using average pooling layers following the depth-wise and separable convolutional layers. As a result, the computational cost was diminished because there were fewer parameters to learn. Each pooling layer was additionally followed by a dropout function with a value of 0.5 to minimize overfitting.

After the final pooling layer, the five CNN branches’ weights were combined in a feature fusion layer, and the output of this layer was used as input to the softmax classification layer to create the output classes. The output classes were left-hand or right-hand movement in one test and left-hand or right-hand imagined movement in another test on *eegmmidb* dataset. For the BCI Competition IV 2a dataset, the output classes were left hand, right hand, feet, and tongue imagined movement. For the BCI Competition IV 2b dataset, the output classes were left-hand and right-hand imagined movement.

### 2.3. Implementation of the Models

The Python TensorFlow framework was used to implement the neural networks. Google Colab GPU was used for testing and training the model. The implementation of this model is publicly available at GitHub (Available online: https://github.com/radia-rayan-chowdhury/EEGNet-Fusion-V2, accessed date: 11 July 2023).

### 2.4. Training and Testing Strategy

The PhysioNet EEG Motor Movement/Imagery dataset contains 45 trials per participant and 360 labeled samples per subject after preprocessing. A total of 37,080 samples from the executed and imagined task subsets for all 103 individuals are labeled. In total, 70% random data were used for training, 10% for validation, and 20% for testing. Each experiment used the same data split because a fixed seed value was applied.

The BCI IV-2a dataset consists of 288 trials and 2 sessions per subject. In this dataset, one session was used for training. The other session was used for testing. In the BCI Competition 2b dataset, there are 5 sessions. Three sessions were used for training. The other 2 sessions were used for testing.

The validation loss was calculated and the model weights with the best validation accuracies were saved during the training period. The model weights were loaded, and during the testing phase, the model was evaluated on the testing dataset by predicting target labels and determining the accuracy. Also, the precision, recall, f1-score and computational time per sample were calculated for the model. The Adam algorithm, a binary cross-entropy loss function, and a learning rate of 0.00001 were applied for optimization. All dropout layers have a dropout probability value of 0.5.

### 2.5. Performance Measure

Accuracy, precision, recall, and f1-score score were measured to examine the model. No negative trials were found in the datasets since all movements were treated as positive trials. The two-class classification required distinct computations for each hand. In one set of assessments, the left-hand (L) target label served as positive, and the right-hand (R) label served as negative. This was used to assess the model’s capacity to differentiate between left- and right-hand movements. The right-hand target label served as a positive value in another set of assessments. If the prediction is L (or R) and the actual label is L (or R), then the value is true positive (TP). If the prediction is L (or R) but the actual label is R (or L), then it is false positive (FP). If the prediction is R (or L), and the actual label is also R (or L), then it is true negative (TN). If the prediction is R (or L), but the actual label is L (or R), then the value is false negative (FN).

Moreover, there are four classes in the BCI IV-2a dataset, and the performance of each class (left-hand (L), right-hand (R), feet (F), and tongue (T)) was calculated separately. At first, the left-hand (L) target label was used as a positive, and the rest of the labels were used as negatives. Similarly, for each time, one class target label was used as positive and the others were used as negative to calculate the accuracy, precision, recall, and F1-score. More comprehensive information can be found in Appendix A.

## 3. Results

This section describes the experimental setup, results, and performance of the proposed model and four benchmark models used for comparison.

### 3.1. Experimental Setup

The PhysioNet EEG Motor Movement/Imagery dataset was tested on our proposed EEGNet Fusion V2, EEGNet Fusion [35], EEGNet [34], MMCNN [46], ShallowConvNet, and DeepConvNet [33]. The dataset was used to evaluate different parameters on EEGNet Fusion V2 model. The dataset also used to evaluate four-branch, six-branch, and seven-branch EEGNet Fusion models. The results were recorded for comparison. For each experiment, 20% of samples were selected at random for testing, 10% for validation, and 70% for training.

Furthermore, the BCI Competition IV-2a and the BCI Competition IV-2b dataset were implemented to measure the performance. The sessions with class labels were used to train the models, and the other sessions were used to test the models for both dataset.

### 3.2. Experimental Results: Evaluation of Different Parameters on EEGNet Fusion V2

In this study, we explored the effect of different filter sizes on the model’s performance. We varied the filter sizes of the convolutional layers in each branch and assessed their impact on the accuracy and computation time of the EEGNet Fusion V2 model. Our results showed that certain filter sizes were more effective in capturing temporal patterns in the EEG signals.

Each time the model was evaluated with different filter sizes of the convolutional layers, the kernel sizes and filter sizes of the separable convolutional layers remained unchanged. Each branch has a 2-D convolutional layers with kernel sizes 8, 16, 32, 64, and 128. A separable convolutional layer was added to each branch with kernel size 16, 32, 64, 128, and 256 and filter size (1, 8), (1, 16), (1, 32), (1, 64), and (1, 128).

The kernel sizes and filter sizes of the separable convolutional layers remained unchanged during each evaluation of the model. For each branch, the kernel sizes were set to 16, 32, 64, 128, and 256, along with filter sizes of (1, 8), (1, 16), (1, 32), (1, 64), and (1, 128). Each time the model was evaluated with different filter sizes for 2-D convolutional layers, the kernel sizes remained unchanged at 8, 16, 32, 64, and 128.

To evaluate the model, we conducted five tests, with each test utilizing different filter sizes for the convolutional layers in each branch. The specific filter sizes employed in each test were as follows:

- Test 1: (1, 64), (1, 128), (1, 256), (1, 512), (1, 1024);
- Test 2: (1, 64), (1, 256), (1, 544), (1, 512), (1, 1024);
- Test 3: (1, 64), (1, 304), (1, 544), (1, 784), (1, 1024);
- Test 4: (1, 64), (1, 80), (1, 96), (1, 112), (1, 128);
- Test 5: (1, 64), (1, 96), (1, 128), (1, 192), (1, 256).

The PhysioNet EEG Motor Movement/Imagery dataset was used to test these models.

Based on the obtained results from Table 1 and Table 2, it is evident that the filter sizes have minimal impact on accuracy but significantly affect the computation time. Therefore, we have made the decision to select the smallest filter sizes (Test 4) for our model. By choosing smaller filter sizes, we aim to optimize the computational efficiency of our model while maintaining a comparable level of accuracy. This allows us to reduce the computational burden and improve the overall efficiency of the model without compromising its performance.

### 3.3. Experimental Results: Evaluation of Different Numbers of EEGNets

To evaluate the proposed five-branch EEGNet Fusion model, we implemented four-branch, six-branch, and seven-branch EEGNet Fusion models. These models share a similar structure to the proposed five-branch model, but with some variations in the number of branches and the kernel and filter sizes.

By implementing these alternative fusion models, we aim to compare their performance with the proposed five-branch model. This comparative analysis enables us to assess the impact of the number of branches and the specific kernel and filter sizes on the overall performance of the EEGNet Fusion architecture. Appendix B provides a comprehensive overview of the filter and kernel sizes utilized in the four-branch, five-branch, six-branch, and seven-branch models.

The PhysioNet EEG Motor Movement/Imagery dataset was used to test these models.

Due to limitations with the available GPU resources in Google Colab, we were unable to implement the higher branch fusion networks to evaluate our proposed model. However, despite this limitation, we proceeded with evaluating our proposed five-branch EEGNet Fusion model and comparing it to the implemented four-branch, six-branch and seven-branch models, while the evaluation could have been more comprehensive with the inclusion of the higher branch models, the results obtained from the four-branch, six-branch, and seven-branch models still provide valuable insights into the performance and effectiveness of the proposed model. Future work could involve conducting evaluations with higher branch fusion networks when more substantial computational resources are available to further investigate the impact of increasing the number of branches on the performance of the EEGNet Fusion architecture.

According to the observations from Table 3 and Table 4, the accuracy generally increases as the number of branches increases. Specifically, there is a noticeable improvement in accuracy when going from three branches to four branches and from four branches to five branches. However, the accuracy of the five branch, six branch and seven-branch models are very close, despite the six branch and seven branch model requiring a significantly higher computation time per sample. Based on these findings, we have decided to consider the five branch model as the proposed model. The five branch model provides a good balance between the accuracy and computation time compared to the six and seven branch models.

### 3.4. Experimental Results: Evaluation with Benchmark Model

#### 3.4.1. Results of PhysioNet EEG Motor Movement/Imagery Dataset

As we improved the EEGNet Fusion model [35], the EEGNet Fusion model was replicated and validated the results on the EEG Motor Movement/Imagery dataset. The paper reported that the model achieved 84.1% and 83.8% accuracy in motor movement and imagined movement task, respectively. Similarly, the replicated model of EEGNet Fusion achieved 84.1% and 84.3% accuracy in motor movement and imagined movement task, respectively, which validated the results of the model.

Table 5 contains the testing accuracy, precision (left and right hand), recall (left and right hand), f1-score (left and right hand), and computing time (per sample) for executed movement tasks from the EEG Motor Movement/Imagery dataset for all examined models. Our proposed EEGNet Fusion V2 model outperformed the other classifiers, and achieved 89.6% accuracy, 89.7% left-hand F1-score, and 89.6% right-hand F1-score. However, the average computation time of the EEGNet Fusion V2 model was 361 ms, which was the slowest and 3.4 times higher than EEGNet Fusion model.

For all evaluated models, Table 6 shows the testing accuracy, precision, recall, f1-score, and computing time (per sample) for imagined movement tasks from the EEG Motor Movement/Imagery dataset. The EEGNet Fusion V2 model performed better than the benchmark models and scored 87.8% accuracy, 87.8% f1-score for left-hand and right-hand, though the computational time per sample was higher than other models, which was 354 ms.

#### 3.4.2. Results of BCI IV-2a Dataset

According to Table 7, the proposed EEGNet Fusion V2 model achieved higher accuracy on BCI IV-2a dataset, which confirmed that the multi-branch architecture outperformed on cross-subject classification. The proposed model scored 74.3% accuracy and took 815 ms computational time per sample. The fastest model, ShallowConvNet, achieved 69.8% accuracy with 44 ms computational time per sample.

#### 3.4.3. Results of BCI IV-2b Dataset

Table 8 demonstrates the accuracy, precision (left and right hand), recall (left and right hand), and f1-score (left and right hand) of all the models on BCI IV-2b dataset. Our EEGNet Fusion V2, outperformed using this dataset and achieved 84.1% with 326 ms computational time per sample.

**Figure 2:**
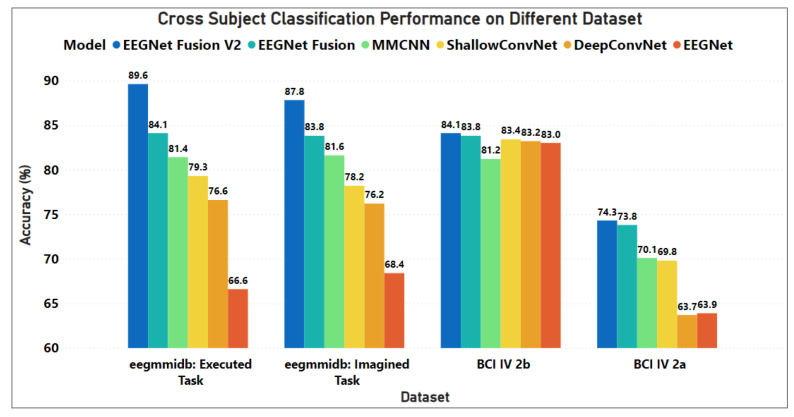
Performance of the models based on EEG Motor Movement/Imagery, BCI Competition IV-2a and IV-2b Dataset. This figure illustrates the proposed EEGNet Fusion V2 model improved the accuracy for these dataset.

The comparison between the EEGNet Fusion V2 model and the benchmark models based on EEG Motor Movement/Imagery, BCI Competition IV-2a and IV-2b dataset were plotted clearly in Figure 2.

Based on these findings, it can be concluded that for cross-subject EEG motor imagery categorization, the EEGNet Fusion V2 model outperforms the other architectures.

## 4. Discussion

The purpose of this research was to increase the accuracy of motor imagery classification to assist people with insufficient motor abilities in communicating with their surroundings through a brain–computer interface. We proposed a multi-branch (five-branch) composite two-dimensional neural network in this study, named the EEGNet Fusion V2 model, to classify the EEG-based motor imagery data. The proposed model was trained and tested on the PhysioNet EEG Motor Movement/Imagery dataset, the BCI Competition IV-2a, and the BCI Competition IV-2b datasets.

The Motor Movement/Imagery dataset was implemented to categorize actual and imagined movements made with the left and right hands. Band-pass signal filtering and a moving window technique were used to preprocess the 103-subject data in this dataset. The EEGNet Fusion V2 model achieved 89.6% and 87.8% accuracy for executed and imagined movements, respectively. In comparison, the EEGNet Fusion model achieved lower accuracy rates of 84.1% for executed and 83.8% for imagined movements. The accuracy for executed movements increased by 5.5%, while the accuracy for imagined movements increased by 4% in the proposed model. The other benchmark models achieved less than 82% accuracy for both executed and imagined movement tasks. However, the proposed model was slower than the other tested models. It took 361 ms and 354 ms of computational time per sample for the real and imagined movement tasks, respectively. The DeepConvNet, ShallowConvNet, MMCNN, EEGNet, and EEGNet Fusion models had computation times of 44 ms, 23 ms, 102 ms, 34 ms, and 107 ms, respectively, for the executed task and 36 ms, 24 ms, 102 ms, 36 ms, and 110 ms, respectively, for the imagined task. These values indicate that the proposed model’s computation time is higher than the other models. Although this computing time was more than three times longer than the EEGNet Fusion model, the computational cost was moderated by the notably greater accuracy of the EEGNet Fusion V2 model.

The BCI Competition IV-2a dataset was implemented to classify four classes: left hand, right hand, feet, and tongue. The BCI Competition IV-2b dataset was also implemented to classify two classes of left and right hands. The proposed model achieved higher accuracy on both datasets, with 74.3% and 84.1% accuracy, respectively. In comparison, the EEGNet fusion model scored 73.8% and 83.8% on the BCI Competition IV-2a and IV-2b datasets, which is less than the proposed model’s performance. The performance of the other benchmark models is below 71% for the IV-2a dataset and 83.5% for the IV-2b dataset.

In our study, we investigated the performance of EEGNet Fusion models with different numbers of branches, specifically four, five, six, and seven. We also explored different filter sizes within the architecture to assess the models. Our findings demonstrate that the five-branch EEGNet Fusion model achieved the highest accuracy with the lowest computational time, outperforming the other models with four, six, and seven branches. As a result, we proposed the five-branch EEGNet Fusion V2 model as the optimal choice due to its performance and less complex structure than the higher-branch models. Our study provides valuable insights into the design of EEGNet Fusion models and offers guidance for future research in this area.

The performance indicates that this composite network outperforms EEGNet Fusion, EEGNet, MMCNN, ShallowConvNet, and DeepConvNet. The results also show that the higher number of multi-branch fusion models obtain higher accuracy and require longer computational time. The key contribution of this study is the examination of multi-branch (five-branch) composite networks using EEG motor imagery data, which represents an enhanced version of EEGNet Fusion. The adaptability of the architecture’s multiple branches improved the model’s performance. The ideal hyperparameter can be set for each branch, and more sophisticated feature maps are created by combining features in the fusion layer. The EEGNet Fusion V2 network can mitigate the complexity of cross-subject EEG classification by providing more flexibility in choosing the hyperparameters.

When developing real-time applications such as brain–computer interfaces (BCIs), it is crucial to prioritize computational efficiency to ensure rapid response following user actions. Otherwise, delays in feedback could lead to a sense of loss of control for the user. As the current model has demonstrated relatively longer computational time when implementing multiple branches, a potential avenue for future research could be to explore strategies for reducing computation time in such scenarios. The exploration of diverse transfer learning approaches can lead to decreased computation time and superior model performance [47,48,49,50]. Furthermore, the computation time can vary significantly depending on the hardware and software used. In this study, the computation time per sample is provided as a reference.

This model can be applied to various EEG-based areas, for example, driver fatigue evaluation, disorder detection, sleep stage, etc. BCI-based motor movement classification can be used to control prosthetic limbs or assistive devices for individuals with limb loss or disabilities. By analyzing brain signals related to motor imagery, therapists can design personalized rehabilitation programs and provide real-time feedback to patients, facilitating motor recovery. Real-time motor movement classification can enable more immersive and interactive gaming experiences where users can perform actions or navigate through virtual worlds by simply imagining the movements.

The proposed model shows strong results when applied to cross-subject classification across the mentioned datasets. However, using the model in real-time scenarios presents challenges due to the dynamic nature of brain signals. The model’s performance might deteriorate when it encounters new subject data that it has not been exposed to before, even though it performs well in controlled experiments. Adapting the model to these changes requires strategies like transfer learning [47,48,49,50], where the model builds upon its existing knowledge to handle new scenarios.

There may be further opportunities to explore more multi-branch designs. As EEG data is non-stationary, which has implications for the feature extraction process, different approaches can be explored to address the issue of non-stationarity [51,52,53]. Transfer learning approaches can be implemented to resolve the issue of less data per subject and inter-subject variations of the EEG-based dataset.

## Figures and Tables

**Table 1:** Evaluation of different parameters on EEGNet Fusion V2 model: results for motor movement executed task.

Test	Accuracy %	Computation Time (per Sample)
Test 1	89.8%	752 ms
Test 2	87.9%	824 ms
Test 3	89.9%	875 ms
Test 4	89.6%	361 ms
Test 5	89.5%	419 ms

**Table 2:** Evaluation of different parameters on EEGNet Fusion V2 model: results for imagery motor movement task.

Test	Accuracy %	Computation Time (per Sample)
Test 1	88.3%	754 ms
Test 2	86.7%	846 ms
Test 3	86.8%	877 ms
Test 4	87.8%	354 ms
Test 5	89.2%	434 ms

**Table 3:** Evaluation of different numbers of EEGNets: results for motor movement executed task.

Model	Accuracy %	Computation Time (per Sample)
3-branch EEGNet	84.1%	107 ms
4-branch EEGNet	85.2%	249 ms
5-branch EEGNet	89.6%	361 ms
6-branch EEGNet	89.8%	555 ms
7-branch EEGNet	89.9%	601 ms

**Table 4:** Evaluation of different numbers of EEGNets: results for imagery motor movement task.

Model	Accuracy %	Computation Time (per Sample)
3-branch EEGNet	83.8%	110 ms
4-branch EEGNet	85.1%	253 ms
5-branch EEGNet	87.8%	354 ms
6-branch EEGNet	87.9%	547 ms
7-branch EEGNet	88.2%	598 ms

**Table 5:** Results for executed motor movement task.

Model	Accuracy %	Precision %		Recall %		F1-Score %		Comp Time (per Sample)
		Left	Right	Left	Right	Left	Right	
DeepConvNet	76.6	76.2	77.1	77.3	76.0	76.7	76.5	44 ms
ShallowConvNet	79.3	79.2	79.3	79.2	79.3	79.2	79.3	23 ms
MMCNN	81.4	82.2	80.6	80.5	82.3	81.3	81.4	102 ms
EEGNet	66.6	69.1	64.8	59.9	73.3	64.2	68.8	34 ms
EEGNet Fusion	84.1	84.2	84.5	83.8	83.9	84.0	84.2	107 ms
**EEGNet Fusion V2**	**89.6**	**89.9**	**89.4**	**89.4**	**89.8**	**89.7**	**89.6**	**361 ms**

The bold texts represent the results of the proposed model.

**Table 6:** Results for imagery motor movement task.

Model	Accuracy %	Precision %		Recall %		F1-Score %		Comp Time (per Sample)
		Left	Right	Left	Right	Left	Right	
DeepConvNet	76.2	76.5	75.9	76.0	76.4	76.3	76.1	36 ms
ShallowConvNet	78.2	78.2	78.3	78.7	77.8	78.5	78.0	24 ms
MMCNN	81.6	81.7	81.5	81.9	81.2	81.8	81.3	102 ms
EEGNet	68.4	68.3	68.4	69.2	67.5	68.8	67.9	36 ms
EEGNet Fusion	83.8	85.0	83.3	82.9	84.8	83.9	84.0	110 ms
**EEGNet Fusion V2**	**87.8**	**88.1**	**87.5**	**87.5**	**88.1**	**87.8**	**87.8**	**354 ms**

The bold texts represent the results of the proposed model.

**Table 7:** Results on BCI Competition IV-2a dataset.

Model	Accuracy %	Precision %				Recall %				F1-Score %				Comp Time
		L	R	F	T	L	R	F	T	L	R	F	T	
DeepConvNet	63.7	67.2	53.8	69.6	73.2	64.5	81.2	56.9	52.3	65.8	64.7	62.6	61.0	51 ms
Shallow ConvNet	69.8	71.6	69.0	70.9	67.6	69.1	72.5	69.6	67.7	70.3	70.7	70.2	67.7	44 ms
MMCNN	70.1	69.4	70.7	72.3	68.3	75.6	72.2	63.7	68.8	72.4	71.5	67.8	68.6	199 ms
EEGNet	63.9	63.8	62.8	62.7	65.9	67.3	63.4	59.3	65.4	65.5	63.1	61.0	65.7	55 ms
EEGNet Fusion	73.8	75.2	73.1	72.8	74.2	76.1	75.0	70.1	74.1	75.6	74.0	71.4	74.1	211 ms
**EEGNet Fusion V2**	**74.3**	**75.0**	**71.1**	**74.7**	**77.0**	**76.5**	**78.2**	**70.7**	**71.9**	**75.8**	**74.5**	**72.6**	**74.4**	**815 ms**

The bold texts represent the results of the proposed model.

**Table 8:** Results on BCI Competition IV-2b dataset.

Model	Accuracy %	Precision %		Recall %		F1-Score %		Comp Time (per Sample)
		Left	Right	Left	Right	Left	Right	
DeepConvNet	83.2	83.1	83.4	83.5	83.0	83.3	83.2	51 ms
ShallowConvNet	83.4	82.6	84.3	84.7	82.1	83.6	83.2	16 ms
MMCNN	81.2	82.9	79.6	78.6	83.7	80.7	81.6	59 ms
EEGNet	83.0	82.5	83.4	83.7	82.3	83.1	82.8	24 ms
EEGNet Fusion	83.8	83.6	83.9	84.0	83.5	83.8	83.7	83 ms
**EEGNet Fusion V2**	**84.1**	**84.7**	**83.6**	**83.3**	**84.9**	**84.0**	**84.2**	**326 ms**

The bold texts represent the results of the proposed model.

## Data Availability

The datasets are publicly available online: https://www.bbci.de/competition/iv/ (accessed date: 15 October 2022) and https://physionet.org/content/eegmmidb/1.0.0/ (accessed date: 22 September 2022).

## Abbreviations

The following abbreviations are used in this manuscript:

MDPI	Multidisciplinary Digital Publishing Institute
DOAJ	Directory of open access journals
TLA	Three letter acronym
LD	Linear dichroism

## Appendix A

The BCI IV-2a dataset includes four classes: left-hand (L), right-hand (R), feet (F), and tongue (T). Each class was assessed separately using metrics like precision, recall, and F1-score. Positive target labels were assigned to one class at a time, while the rest were treated as negative.

For the left-hand (L) class:

- TP: Prediction is L and actual label is L.
- FP: Prediction is L but actual label is R, F, or T.
- TN: Prediction is R, F, or T, and actual label is also R, F, or T.
- FN: Prediction is R, F, or T, but actual label is L.

For the right-hand (R) class:

- TP: Prediction is R and actual label is R.
- FP: Prediction is R but actual label is L, F, or T.
- TN: Prediction is L, F, or T, and actual label is also L, F, or T.
- FN: Prediction is L, F, or T, but actual label is R.

For the feet (F) class:

- TP: Prediction is F and actual label is F.
- FP: Prediction is F but actual label is L, R, or T.
- TN: Prediction is L, R, or T, and actual label is also L, R, or T.
- FN: Prediction is L, R, or T, but actual label is F.

For the tongue (T) class:

- TP: Prediction is T and actual label is T.
- FP: Prediction is T but actual label is L, R, or F.
- TN: Prediction is L, R, or F, and actual label is also L, R, or F.
- FN: Prediction is L, R, or F, but actual label is T.

## Appendix B

To assess the proposed five-branch EEGNet Fusion V2 model, we conducted experiments using four-branch, six-branch, and seven-branch EEGNet Fusion models. The filter and kernel sizes implemented in these models are listed below.

The four-branch EEGNet:

- Convolutional Filter sizes for each branch: (1, 64), (1, 80), (1, 96), and (1, 112)
- Convolutional Kernel sizes for each branch: 8, 16, 32, and 64
- Separable Filter sizes for each branch: (1, 8), (1, 16), (1, 32), and (1, 64)
- Separable Kernel sizes for each branch: 16, 32, 64, and 128

The proposed five-branch EEGNet:

- Convolutional Filter sizes for each branch: (1, 64), (1, 80), (1, 96), (1, 112), and (1, 128)
- Convolutional Kernel sizes for each branch: 8, 16, 32, 64, and 128
- Separable Filter sizes for each branch: (1, 8), (1, 16), (1, 32), (1, 64), and (1, 128)
- Separable Kernel sizes for each branch: 16, 32, 64, 128, and 256

The six-branch EEGNet:

- Convolutional Filter sizes for each branch: (1, 32), (1, 64), (1, 80), (1, 96), (1, 112), and (1, 128)
- Convolutional Kernel sizes for each branch: 4, 8, 16, 32, 64, and 128
- Separable Filter sizes for each branch: (1, 8), (1, 16), (1, 32), (1, 64), (1, 128) and (1, 256)
- Separable Kernel sizes for each branch: 8, 16, 32, 64, 128, and 256

The seven-branch EEGNet:

- Convolutional Filter sizes for each branch: (1, 16), (1, 32), (1, 64), (1, 80), (1, 96), (1, 112), and (1, 128)
- Convolutional Kernel sizes for each branch: 2, 4, 8, 16, 32, 64, and 128
- Separable Filter sizes for each branch: (1, 4), (1, 8), (1, 16), (1, 32), (1, 64), (1, 128) and (1, 256)
- Separable Kernel sizes for each branch: 4, 8, 16, 32, 64, 128, and 256
